# Supplementary material for: Association between oral microbiome and seven types of cancers in East Asian population: a two-sample Mendelian randomization analysis
Source: Front Mol Biosci. 2023 Nov 21;10:1327893. doi: 10.3389/fmolb.2023.1327893 (PMC10702768; doi:10.3389/fmolb.2023.1327893)
Supplement: Supplementary file 1 [file DataSheet1.ZIP › Supplementary Materials/MR plots of saliva/saliva═╝/Lung Cancer/pheno.662_to_lung cancer_forest.pdf]

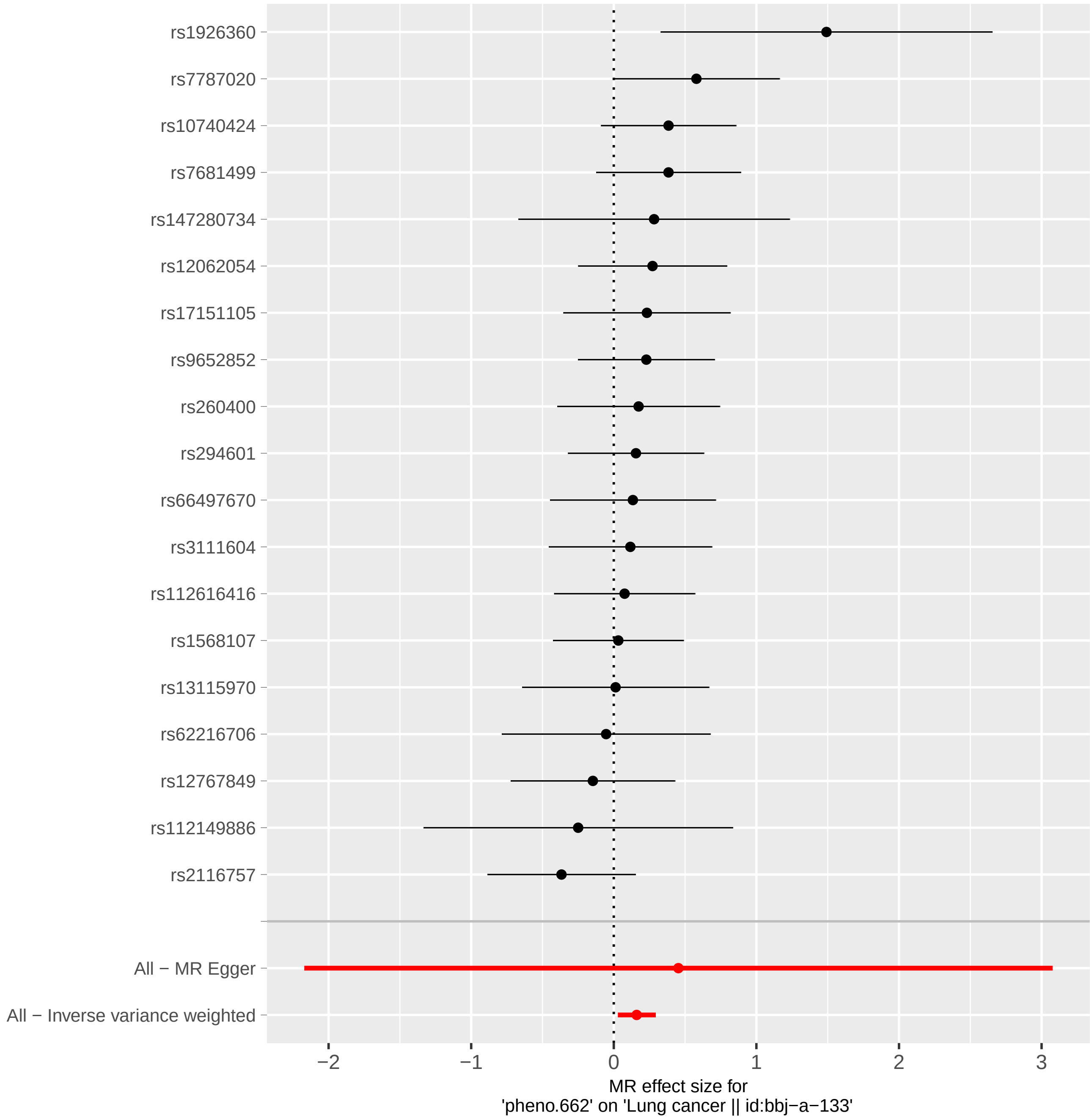
rs1926360
rs7787020
rs10740424
rs7681499
rs147280734
rs12062054
rs17151105
rs9652852
rs260400
rs294601
rs66497670
rs3111604
rs112616416
rs1568107
rs13115970
rs62216706
rs12767849
rs112149886
rs2116757
All − MR Egger
All − Inverse variance weighted
−2
−1
0
1
2
3
MR effect size for
'pheno.662' on 'Lung cancer || id:bbj−a−133'
